# Differential binding affinity of mutated peptides for MHC class I is a predictor of survival in advanced lung cancer and melanoma

**DOI:** 10.1093/annonc/mdx687

**Published:** 2017-10-23

**Authors:** E Ghorani, R Rosenthal, N McGranahan, J L Reading, M Lynch, K S Peggs, C Swanton, S A Quezada

**Affiliations:** 1 Cancer Immunology Unit, University College London Cancer Institute, London, UK; 2 Cancer Research UK Lung Cancer Centre of Excellence, University College London, London, UK; 3 Translational Cancer Therapeutics Laboratory, The Francis Crick Institute, London, UK;; 4 Department of Biostatistics and Bioinformatics, Roswell Park Cancer Institute, Buffalo, USA

**Keywords:** peptide immunogenicity, neoantigen prediction, immunoinformatics, immunotherapy

## Abstract

**Background:**

Cancer mutations generate novel (neo-)peptides recognised by T cells, but the determinants of recognition are not well characterised. The difference in predicted class I major histocompatibility complex (MHC-I) binding affinity between wild-type and corresponding mutant peptides (differential agretopicity index; DAI) may reflect clinically relevant cancer peptide immunogenicity. Our aim was to explore the relationship between DAI, measures of immune infiltration and patient outcomes in advanced cancer.

**Patients and methods:**

Cohorts of patients with advanced non-small-cell lung cancer (NSCLC; LUAD, *n *=* *66) and melanoma (SKCM, *n *=* *72) were obtained from The Cancer Genome Atlas. Three additional cohorts of immunotherapy treated patients with advanced melanoma (total *n *=* *131) and NSCLC (*n *=* *31) were analysed. Neopeptides and their clonal status were defined using genomic data. MHC-I binding affinity was predicted for each neopeptide and DAI values summarised as the sample mean DAI. Correlations between mean DAI and markers of immune activity were evaluated using measures of lymphocyte infiltration and immune gene expression.

**Results:**

In univariate and multivariate analyses, mean DAI significantly correlated with overall survival in 3/5 cohorts, with evidence of superiority over nonsynonymous mutational and neoantigen burden. In these cohorts, the effect was seen for mean DAI of clonal but not subclonal peptides. In SKCM, the association between mean DAI and survival bordered significance (*P* = 0.068), reaching significance in an immunotherapy-treated melanoma cohort (*P* = 0.003). Mean DAI but not mutational nor neoantigen burden was positively correlated with independently derived markers of immune infiltration in both SKCM (*P *=* *0.027) and LUAD (*P *=* *0.024).

**Conclusions:**

The association between mean DAI, survival and measures of immune activity support the hypothesis that DAI is a determinant of cancer peptide immunogenicity. Investigation of DAI as a marker of immunologically relevant peptides in further datasets and future clinical studies of neoantigen based immunotherapies is warranted.

## Introduction

Cancer mutations encode novel (neo-)peptides that can be presented to T cells by major histocompatibility complex (MHC) molecules. A subset of neopeptides are sufficiently self-dissimilar to be immune targets and are termed neoantigens. These can render individual tumours uniquely antigenic [1, 2] by forming a substrate for lymphocyte mediated anti-cancer immunity.

We and others have identified neoantigen reactive T cells in non-small-cell lung cancer (NSCLC) [3], melanoma [4] and gastrointestinal tumours [5], with observational and experimental evidence to suggest their role in clinically relevant tumour control [6, 7].

Based on high-throughput tumour genomic analysis, each nonsynonymous mutation (referred to as mutation, hereafter) can potentially give rise to multiple neopeptides, resulting in a vast total number. Specific identification of immunogenic candidates is consequently a major challenge. Studies of immunity to infectious agents first suggested peptide immunogenicity is associated with affinity for MHC, with strong binders considered more likely to achieve cell surface presentation and thus the opportunity for immune recognition [8].

In support of a relationship between predicted neopeptide affinity and immunogenicity, recent clinical studies have shown a correlation between the burden of strong-binding neopeptides with affinity for MHC-I of <500 nM (referred to as neoantigens) and patient outcome in advanced melanoma and lung cancer [9–11].

However, strategies to improve neopeptide immunogenicity prediction are required, as illustrated by multiple studies revealing T-cell responses to only 0%–3% of predicted strong binders [3, 12, 13] and the recognition of neoantigens of low predicted affinity [5, 14]. Suboptimal performance of affinity prediction algorithms likely contributes to these findings, but the description of immunogenic neopeptides with very low *invitro* measured MHC affinity supports the concept that weak MHC binding alone is insufficient to exclude immunogenicity [15].

We have recently shown that less heterogenous cancers bearing a high burden of clonal neoantigens shared by all cells are more strongly associated with survival than simply those with high neoantigen burden, suggesting neopeptide features other than affinity contribute to patient outcomes [3].

Tumour reactive T cells can differentiate between self and mutant peptides that differ by a single amino acid. Mechanistically, preclinical work suggests the difference in predicted affinity for any given wild-type/mutant peptide pair (termed differential agretopicity index; DAI) is a broad indicator of neopeptide dissimilarity from self and a feature of immunogenicity. For individual peptides in a preclinical model, DAI was reportedly a better indicator of immunogenicity than mutant affinity [15]. Extending this to human tumours, we hypothesised that tumours enriched for high DAI neopeptides may be more susceptible to immune recognition and hence clinically relevant tumour control. As immunogenic strong binding neopeptides are described in both lung cancer [3] and melanoma [4], we additionally hypothesised DAI may be of particular relevance amongst this subset.

Using sequencing data from the Cancer Genome Atlas (TCGA) and three published cohorts of patients with advanced melanoma and lung cancer re-analysed with our peptide affinity prediction pipeline, we investigated the relationship between patient survival, markers of immune activity and DAI, to define whether this measurement is relevant to the human anti-tumour immune response.

## Methods

### Clinical cohorts and outcome assessments

Cohorts of patients with stage III/IV lung adenocarcinoma (LUAD; *n *=* *106/522) and stage IIIC/IV cutaneous melanoma (SKCM; *n *=* *145/470) were identified from TCGA and served as datasets for initial evaluation of the association between DAI on survival. Advanced stage TCGA patients were selected to match immunotherapy-treated cohorts described below. Patients with stage III and IV disease in the LUAD cohort had similar survival outcomes and the former group was therefore included in analyses.

Further datasets of immunotherapy-treated patients comprised a cohort with stage IV NSCLC of predominantly adenocarcinoma subtype treated with pembrolizumab [11] and two cohorts of patients with advanced melanoma treated with anti-CTLA-4 directed immunotherapies [9, 10].

Following filtering to retain high-quality samples (see supplementary Methods, available at *Annals of Oncology* online), final cohorts consisted of *n *=* *66 LUAD, *n *=* *75 SKCM, *n *=* *78 Van Allen, *n *=* *31 Rizvi and *n *=* *51 Snyder patients.

Patient survival was the primary outcome measure in this study. For TCGA datasets, Snyder [9] and Van Allen [10], overall survival data are available. For the Rizvi cohort [11], progression-free survival only is available.

### Neopeptide prediction and DAI analysis

Full details of the informatics pipeline used to identify patient HLA status, nonsynonymous mutations and the predicted MHC-I binding affinity of mutant peptides have previously been published [3] and outlined in the supplementary Methods, available at *Annals of Oncology* online. Mutation clonality was inferred from single sample sequenced tumours using a modified version of PyClone as previously described.

To calculate DAI, MHC-I affinity was predicted for mutant and wild-type peptide pairs arising from the same mutation and differing by a single amino acid. The DAI of each mutant peptide was calculated by subtraction of its predicted binding affinity from the value of the corresponding wild-type peptide. Further details are within the supplementary Methods, available at *Annals of Oncology* online.

## Results

To assess the relationship between DAI and patient survival in advanced cancer, we selected TCGA and immunotherapy-treated cohorts of patients with advanced lung cancer and melanoma for whom high quality whole exome sequencing and outcome data were available, with demographics summarised in supplementary Table S1, available at *Annals of Oncology* online.

Preclinical work has previously found high DAI peptides to be preferentially mutated at anchor residues [15] and we tested this relationship in human samples. Amongst all 9mer peptides from the LUAD cohort (*n *=* *166 746), we found a strong correlation between probability of anchor residue mutation and DAI predicted for HLA-A, with close to 100% of the most positive and negative DAI peptides mutated at anchor residues P2 and P9 (supplementary Figure S1, available at *Annals of Oncology* online).

**Figure 1:**
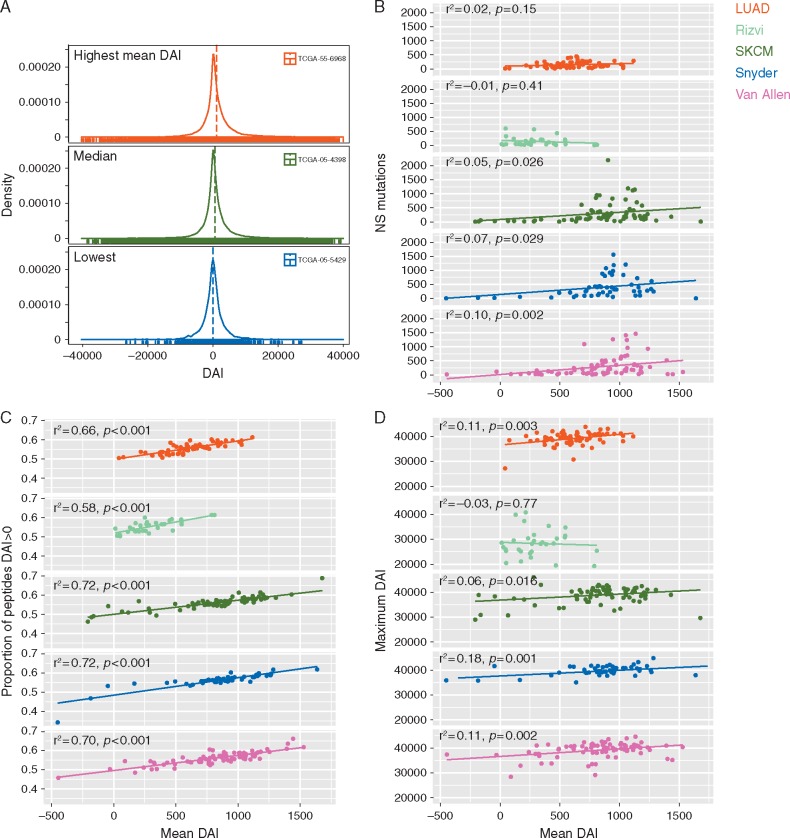
(A) Distribution of DAI for all peptides in three LUAD samples (highest, average and lowest mean DAI, respectively). (B–D) Correlation between mean DAI and non-synonymous (NS) mutation load, proportion of peptides with a DAI >0 and maximum DAI across five cohorts was evaluated by linear regression.

**Figure 2:**
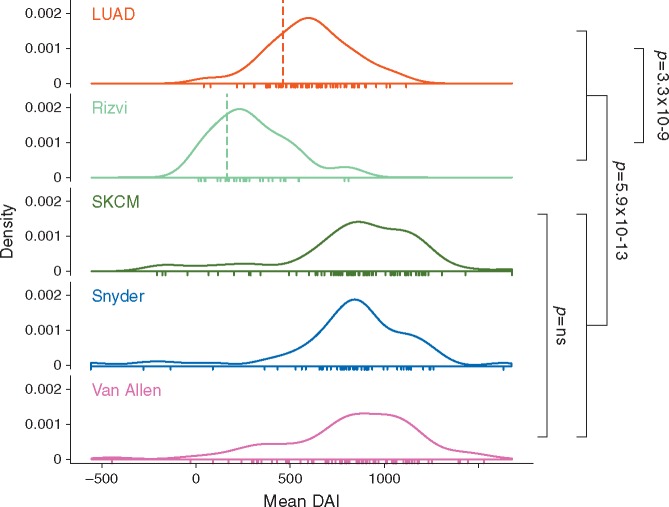
. Density plots representing the distribution of mean DAI across cohorts, with dotted lines indicating the first quartile cut point used to stratify patients for subsequent survival analysis in LUAD and Rizvi lung cancer cohorts. One way ANOVA *P*-values are shown.

Mean DAI was selected to summarise DAI values for each sample. For individual patients and across cohorts, mean DAI was found to associate with both maximum DAI and the proportion of peptides with DAI >0 nM (Figure 1). As an indicator of both DAI magnitude and positive skew, mean DAI therefore represents a suitable indicator of samples enriched for high DAI neopeptides. Whilst mean DAI distribution was similar across melanoma cohorts, LUAD patients had significantly higher values compared with Rizvi [11] (Figure 2; supplementary Table S2, available at *Annals of Oncology* online).

As the relationship between mean DAI and outcomes was non-linear, patients were stratified into mean DAI quartiles for survival analysis. Thresholds of low versus high mean DAI for each cancer type were defined in TCGA data. By subsequently applying these thresholds for outcome analysis in the corresponding immunotherapy-treated cohorts, we tested for a survival signal magnified by treatment.

**Figure 3:**
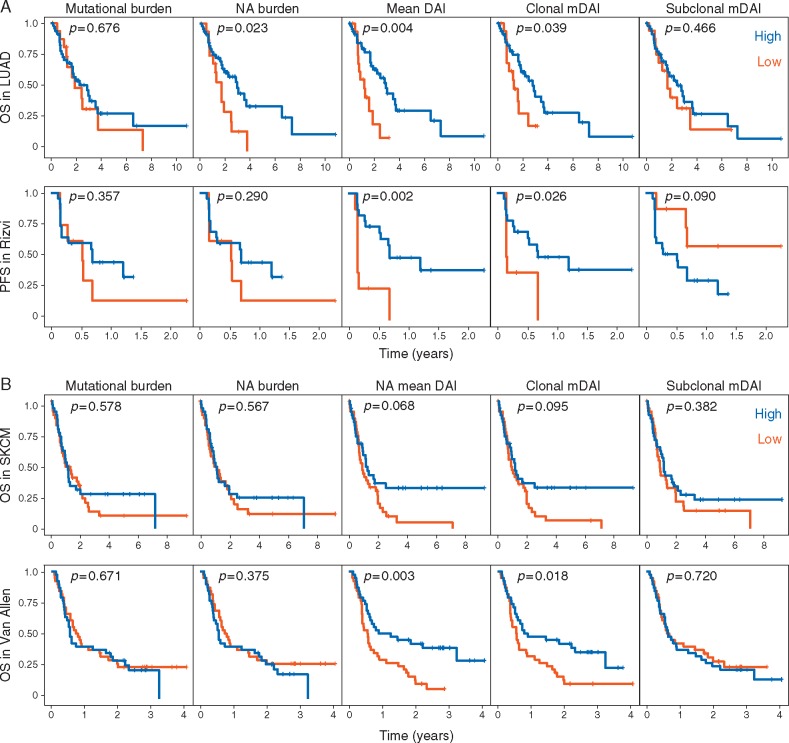
. Kaplan–Meier survival curves for patients with advanced lung cancer (A; TCGA LUAD and Rizvi cohorts) and melanoma (B; TCGA SKCM and Van Allen cohorts), stratified into high and low comparator groups for each variable (columns). (A) Mean DAI was calculated for all predicted neopeptides. For each variable, patients were stratified into high (>first quartile) and low (<first quartile) subgroups, respectively. (B) Mean DAI was calculated for mutant peptides with a predicted MHC affinity of <500 nM (neoantigens). For each variable, patients were stratified into high (>median) and low (<median) subgroups respectively. Mean DAI (mDAI) was additionally calculated for peptides arising from clonal and subclonal mutations. Log rank *P*-values are shown. Mutational and neoantigen burden refers to the number of nonsynonymous mutations and neoantigens, respectively. OS, overall survival; PFS, progression free survival; NA, neoantigen.

In LUAD, cut point analysis by inspection of survival curves and univariate Cox regression (supplementary Figure S2A, available at *Annals of Oncology* online) revealed low mean DAI (≤lower quartile) to significantly associate with worse overall survival (*P *=* *0.004; Figure 3A). The correlation between mean DAI and survival at this threshold was replicated in the Rizvi cohort [11] (*P *=* *0.002).

In comparison, neoantigen but not mutational load was found to predict survival in LUAD (*P *=* *0.023 and 0.675, respectively) and neither were predictive in Rizvi [11] using the same threshold to define low versus high groups.

High-affinity neoantigens are targeted by T cells in both NSCLC [3] and melanoma [4], suggesting peptides with combined high affinity and DAI may be particularly immunogenic. We tested this hypothesis by calculating mean DAI amongst neoantigens of predicted affinity <500 nM. Neoantigen mean DAI was not associated with survival in LUAD (*P *=* *0.66), but was in Rizvi [11] (*P *=* *0.04, supplementary Figure S3, available at *Annals of Oncology* online).

Applying this approach to melanoma, mean DAI of all peptides was not associated with overall survival in SKCM. Excluding low-affinity peptides, we calculated the neoantigen mean DAI. Neoantigen mean DAI was similarly distributed across the melanoma cohorts (supplementary Figure S4 and Table S3, available at *Annals of Oncology* online).

Cut point analysis revealed low neoantigen mean DAI (≤median; supplementary Figure S2B, available at *Annals of Oncology* online) to associate with a non-statistically significant trend to poor overall survival in SKCM (*P *=* *0.068; Figure 3B). Applying the same threshold, we found low neoantigen mean DAI to correlate with poorer survival in the Van Allen [10] (*P *=* *0.003, Figure 3B) but not Snyder cohorts [9] (supplementary Figure S5, available at *Annals of Oncology* online, *P *=* *0.582). Neither neoantigen nor mutational burden correlated with survival in the melanoma cohorts, although tests of association at other thresholds were not carried out.

As affinity prediction values may be most accurate for 9mer peptides, mean DAI was recalculated for 9mers in the LUAD and SKCM cohorts, and was found to correlate better with survival compared with all-mer mean DAI, particularly in the SKCM cohort (supplementary Figure S6, available at *Annals of Oncology* online; all-mer *P *=* *0.069, 9mer *P *=* *0.035).

Low neoantigen intra-tumoural heterogeneity (defined as the proportion of neoantigens derived from subclonal mutations) combined with high neoantigen burden is a superior measure of patient outcome compared with the latter alone and we have additionally shown the immunogenicity of clonal neoantigens [3]. This subset may play an important role in anti-cancer immunity and we therefore next evaluated the association between survival and mean DAI of peptides according to clonality. For cohorts within which mean DAI was associated with survival, this was the case when calculated for neopeptides arising from clonal but not subclonal mutations (Figure 3).

**Figure 4:**
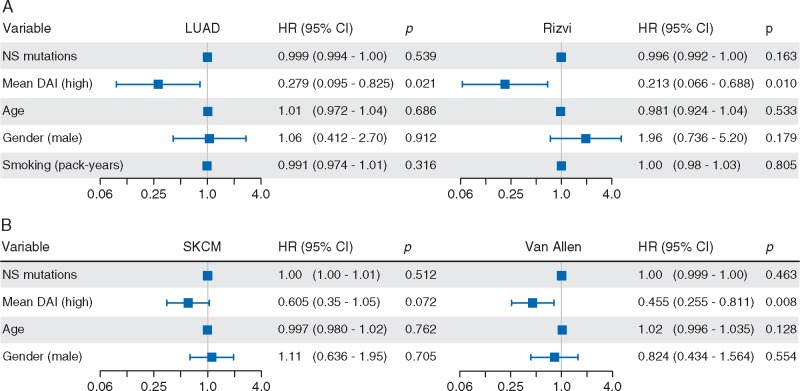
. Multivariate Cox regression modelling of survival in advanced lung cancer (A) and melanoma (B). NS, non-synonymous; NA, neoantigen; HR, hazard ratio; CI, confidence interval. Data on *n* = 74/75 SKCM patients available for analysis.

Mean DAI weakly correlated with mutational burden in melanoma but not lung cancer cohorts (Figure 1) so we carried out multivariate Cox regression to control for potential confounding effects. After correction for multiple factors, the correlation between mean DAI and survival remained significant in LUAD, Rizvi and Van Allen (Figure 4). As a continuous variable, mutational burden did not correlate with survival in the four cohorts tested.

**Figure 5:**
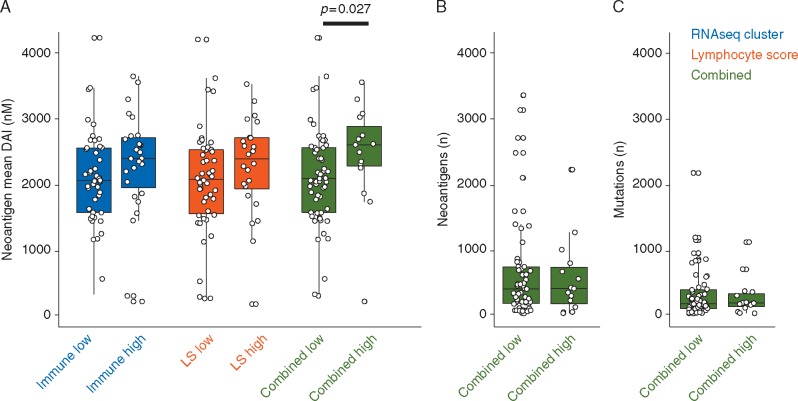
. TCGA patients with advanced melanoma have previously been stratified into high and low immune-infiltrated groups based on unsupervised cluster analysis of transcriptomic data (RNAseq cluster) and histopathological assessment of lymphocyte density and distribution (lymphocyte score, LS). (A) Patients with immune-infiltrated tumours as defined by RNAseq cluster combined with a high LS have a significantly higher neoantigen mean DAI. (B, C) Mutational and neoantigen burden were not different between high- and low-infiltrated groups. Wilcoxon rank sum test *P*-values are shown.

In SKCM, lymphocyte density and distribution were previously measured to define a semi-quantitative lymphocyte score and an RNA expression profile of high immune infiltration was determined [16]. Whilst neoantigen mean DAI did not correlate with high lymphocyte infiltration (score >2 as originally defined) nor immune gene expression when tested individually (Figure 5A), patients with both factors were found to have a significantly higher neoantigen mean DAI (*P *=* *0.027), with no difference in mutational nor neoantigen burden (Figure 5B and C).

**Figure 6:**
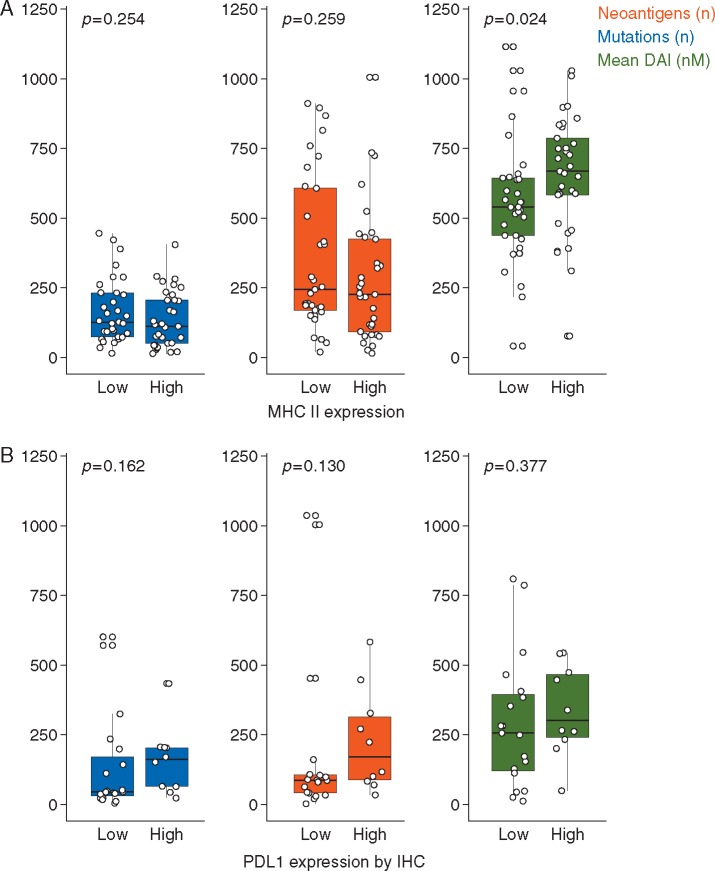
. (A) A 13-gene MHC-II expression signature has previously been shown to correlate with immune infiltration in LUAD. Patients with a high (above the median) MHC-II expression score have higher mean DAI but no difference in mutational/neoantigen burden. (B) Patients in the Rizvi cohort were stratified into high and low PD-L1 expression groups based on previously published histopathological evaluation (*n *=* *29 available for analysis). There is a non-statistically significant trend of association between PD-L1 expression and mutation/neoantigen burden and mean DAI. Wilcoxon rank sum test *P*-values are shown.

In LUAD, a 13 gene MHC-II expression signature has recently been shown to strongly correlate with the presence of multiple MHC-II expressing immune cell types, serving as a proxy measure of infiltration [17]. As tissue MHC-II expression is upregulated by IFN-γ produced during effector T cell activation, this signature may additionally represent a marker of immune activity. Having stratified the cohort by MHC-II expression score based on TCGA RNA-sequencing data, high expression (>median) significantly correlated with mean DAI (*P *=* *0.024), but not mutational nor neoantigen burden (Figure 6A).

In the absence of RNA-sequencing data for the Rizvi dataset, we investigated the relationship between mean DAI and tumour PDL1 expression as an indicator of immune infiltration, and found a non-statistically significant association (Figure 6B).

## Discussion

Multiple factors including peptide abundance, MHC affinity, stability and amino acid composition shape peptide immunogenicity [18]. Self-similar HIV peptides are less likely to be T cell targets [19] and we have recently demonstrated the importance of frameshift insertion and deletion (indel) cancer mutations in generating highly self-dissimilar neoantigens that correlate with immunotherapy efficacy [20]. The notion that peptide self-dissimilarity favours immune recognition was developed in pre-clinical work that defined DAI as an indicator of immunogenicity [15]. In this study, predicted affinity alone as an immunogenicity marker was challenged by the finding that 8/10 immunogenic high DAI peptides had a measured affinity of >500 nM, of which 6 had affinity of >50 000 nM indicative of weak/non-MHC binding by conventional criteria.

Should DAI mark peptide immunogenicity, we reasoned that samples enriched for high DAI peptides may be more susceptible to immune recognition, translating to a survival advantage.

Mean DAI correlates with the proportion of peptides with DAI >0 nM and the sample maximum DAI, which are individually associated with survival in independent cohorts (data not shown). As mean DAI captures both metrics, it was chosen as a summary score to indicate tumours biased towards high DAI peptides. Assuming peptide synthesis rates are DAI independent, a greater intracellular representation of high DAI neopeptides would be expected in samples enriched for mutations engendering these species. Protein abundance correlates with the probability of MHC presentation [21] and as the capacity of peptide presentation pathways is finite, it is conceivable that presentation of high DAI neopeptides is favoured in samples with positively skewed DAI.

The demonstration that mean DAI correlates with survival in three cohorts of patients across two tumour types supports DAI as a potential contributor to peptide immunogenicity in cancer. Ongoing work is aimed at further refining DAI summary metrics to improve upon the performance of the sample mean and more precisely characterise features of immunogenicity amongst high DAI peptides.

We have previously reported that neoantigen clonal architecture is a likely determinant of the anti-cancer immune response [3]. The finding that mean DAI of clonal but not subclonal mutations is associated with patient survival supports the hypothesis that effective immune responses are directed towards the latter.

Although recent landmark studies correlated mutational/neoantigen burden and clinical outcome, our results are divergent. Van Allen et al. [10] measured a composite clinical outcome, as opposed to overall survival used here. Snyder et al. [9] found overall survival was correlated with high-mutational burden in discovery but not validation sets. In a subsequent re-analysis, the association was limited to patients who underwent tissue biopsy before but not post therapy initiation [22]. In the Rizvi study [11], above median mutational/neoantigen burden correlated with survival, but this association is not apparent here using the lower quartile as a stratification threshold. Neoantigen but not mutational burden was associated with improved survival in LUAD (Figure 3A), arguing against these factors as determinants of survival in advanced NSCLC generally.

Whilst no common threshold defined high mean DAI across the tumour sites studied, our approach of defining thresholds in TCGA data and discovering confirmation in secondary cohorts supports the validity of our findings. The observation that independently described markers of immune infiltration are associated with mean DAI in both SKCM and LUAD supports the hypothesis that DAI may be a marker of peptide immunogenicity.

The suggestion that mean DAI is a better survival predictor in immunotherapy-treated cohorts further supports our conclusions. Although our study lacked power to discover an immunotherapy effect, whilst high neoantigen mean DAI is not a significant survival factor in LUAD nor SKCM, there was a clear significant association in the Rizvi and Van Allen cohorts [10, 11].

Many neopeptides verified to be immunogenic have high predicted MHC affinity, and we hypothesised that mean DAI may be of specific relevance amongst this subset. The finding that mean DAI of high affinity binders correlates with survival is in keeping with the role of this factor in characterising cancer peptide immunogenicity. This effect is more pronounced in melanoma than lung cancer, suggesting the potential immunogenicity of high DAI/low affinity peptides in the latter.

This difference, along with the finding that no common mean DAI threshold separates good from poor prognostic categories across the two cancer types studied, indicates possible context dependency of rules governing peptide immunogenicity. Whilst mean DAI may indicate intra-tumoural differences in T cell antigen recognition, immunosuppressive mechanisms may differ by tumour type and site and are likely to differentially affect the overall immunogenicity of tumour cells and infiltrating T cell effector function. Different thresholds to define clinically significant values of mean DAI may therefore reflect differential activity of regulatory pathways, making it unlikely for the same threshold to apply across tumour types, as in some cases stronger antigenic stimulation may be required to bypass tumour immunosuppression.

A number of clinical trials are investigating neoantigen-based vaccines and target selection is critical to development of successful therapeutics. In one recent phase I study, predicted neoantigens with mutations occurring at MHC anchor residues were prioritised [23]; such mutations generate high DAI neopeptides. Our study provides evidence in favour of this and more direct approaches to selection of high DAI peptides in future trials.

In summary, we have shown that mean DAI is associated with clinical outcome in patients with advanced melanoma and lung cancer. Mean DAI is relevant only amongst clonal mutations and correlates with immunotherapy efficacy and indicators of immune infiltration. Our findings support the notion that DAI is a relevant predictor of neopeptide immunogenicity that should be considered in ongoing attempts to refine the selection of neoantigen targets for adoptive cell transfer and vaccine studies.

## Supplementary Material

Supplementary Figures S1-6

Supplementary Methods and Tables
