# Supplementary material for: Differential binding affinity of mutated peptides for MHC class I is a predictor of survival in advanced lung cancer and melanoma
Source: Ann Oncol. 2017 Oct 23;29(1):271–9. doi: 10.1093/annonc/mdx687 (PMC5834109; doi:10.1093/annonc/mdx687)
Supplement: Supplementary Figures S1-6 [file supplementary_figures_s1-6_mdx687.pdf]

Figure S1. Anchor residues mutations are frequently observed at extreme values of peptide DAI

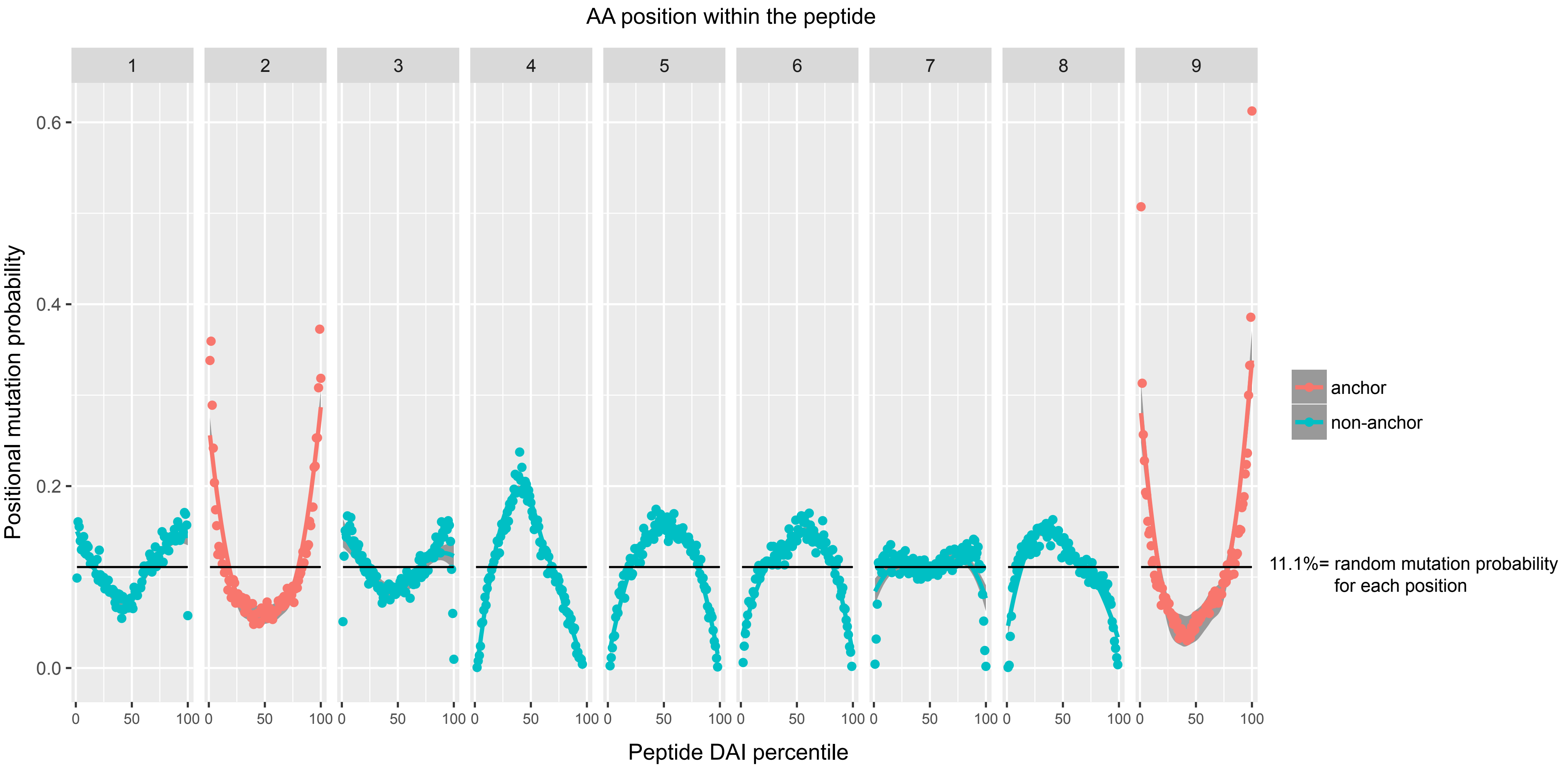

9mer HLA-A binding peptides (n=166746) for patients in the LUAD cohort were ranked according to DAI. At each DAI percentile, the probability of a mutation at each AA position is represented. At strongly positive and negative peptide DAI values, anchor positions 2 and 9 are most likely to be mutated. The expected mutation probability at each position assuming a random distribution (11.1%) is represented with a solid line.

<u>Figure S2. Determination of cutpoints for survival analysis</u>

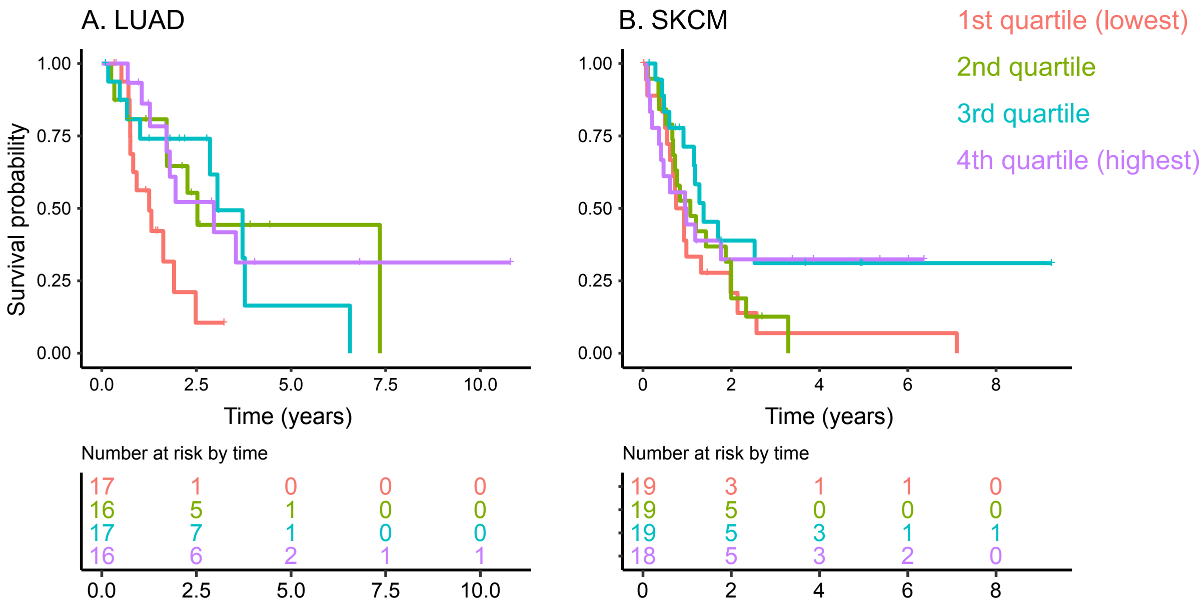

Study	Cutpoint (quartile)	HR	95% CI	*p*-value	Adjusted *p*
**LUAD**	**≤1st**	**0.318**	**0.149-0.679**	**0.003**	**0.009**
	≤2nd	0.549	0.284-1.06	0.074	0.111
	≤3rd	0.787	0.359-1.73	0.551	0.551
**SKCM**	≤1st	0.654	0.369-1.159	0.146	0.219
	**≤2nd**	**0.611**	**0.358-1.043**	**0.071**	**0.213**
	≤3rd	0.866	0.455-1.646	0.660	0.660

(A-B) Survival of subgroups within LUAD and SKCM. Patients were divided into mean DAI (A, LUAD) and neoantigen mean DAI (B, SKCM) quartile subgroups and Kaplan-Meier survival estimates plotted. The visual impression that quartile 1 in LUAD and the median in SKCM serve as cutpoints to divide patients into prognostic categories was tested by univariate Cox regression modelling with Benjamini-Hochberg adjustment of *p*-values (Table). Cutpoints used in further analysis are in bold. HR, hazard ratio; CI, confidence interval.

Figure S3. Neoantigen mean DAI in lung cancer cohorts

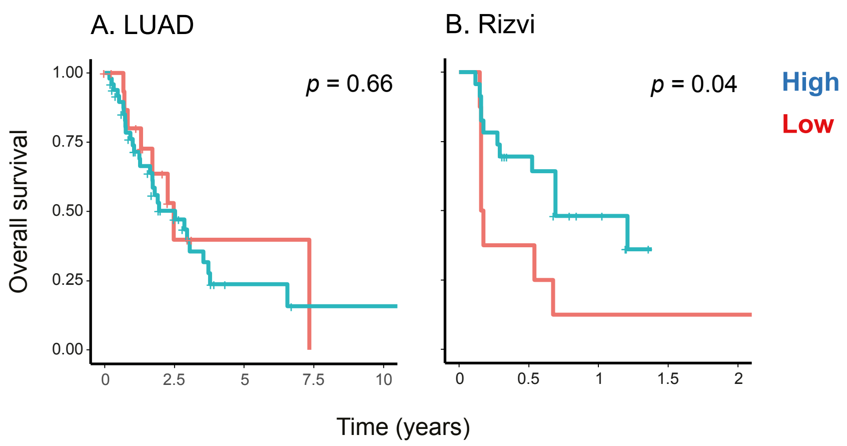

Kaplan-Meier plots of the association between neoantigen mean DAI in (A) LUAD and (B) Rizvi [11] cohorts with survival. Mean DAI was calculated for all peptides with a binding affinity <500 nM and patients stratified into low (first quartile) vs high (upper three quartiles) groups for survival analysis. Log rank *p*-values are shown.

Figure S4. Distribution of neoantigen mean DAI in melanoma cohorts

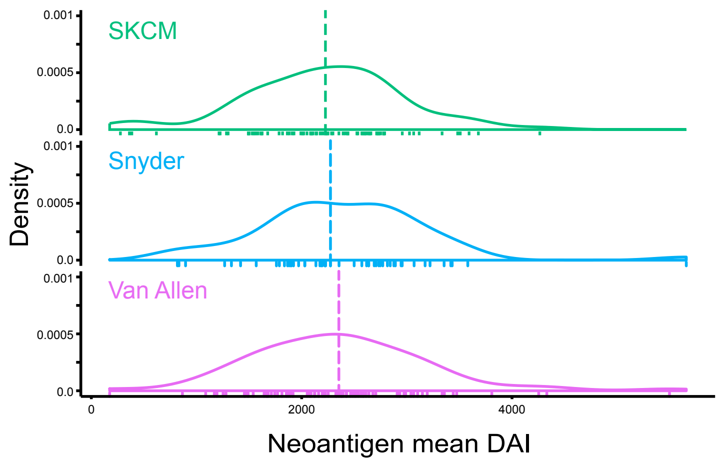

Density plots of mean DAI of peptides with binding affinity <500 nM across SKCM, Snyder [9] and Van Allen [10] melanoma cohorts, with dotted lines indicating the median cutpoint used to stratify patients for subsequent survival analysis.

Figure S5. Predictors of outcome in Snyder

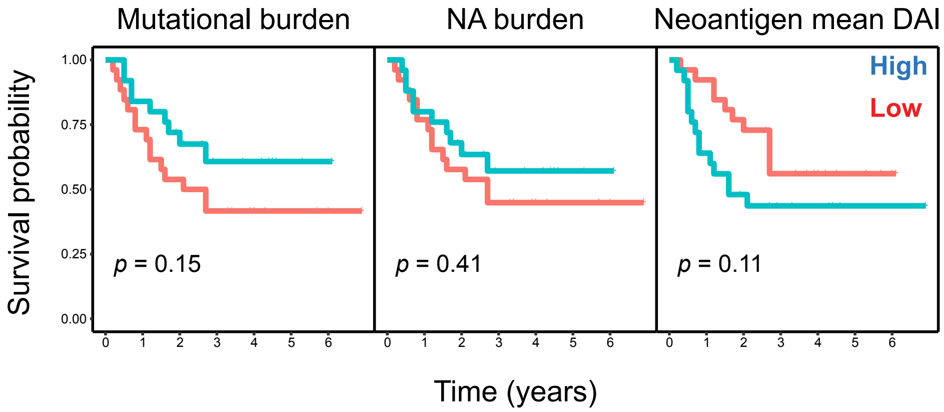

Patients in the Snyder cohort [9] were stratified into high and low groups according to the median value for each factor. Kaplan-Meier survival curves and log rank *p*-values are shown.

Figure S6. Association between survival and mean DAI calculated for 9mers in LUAD and SKCM

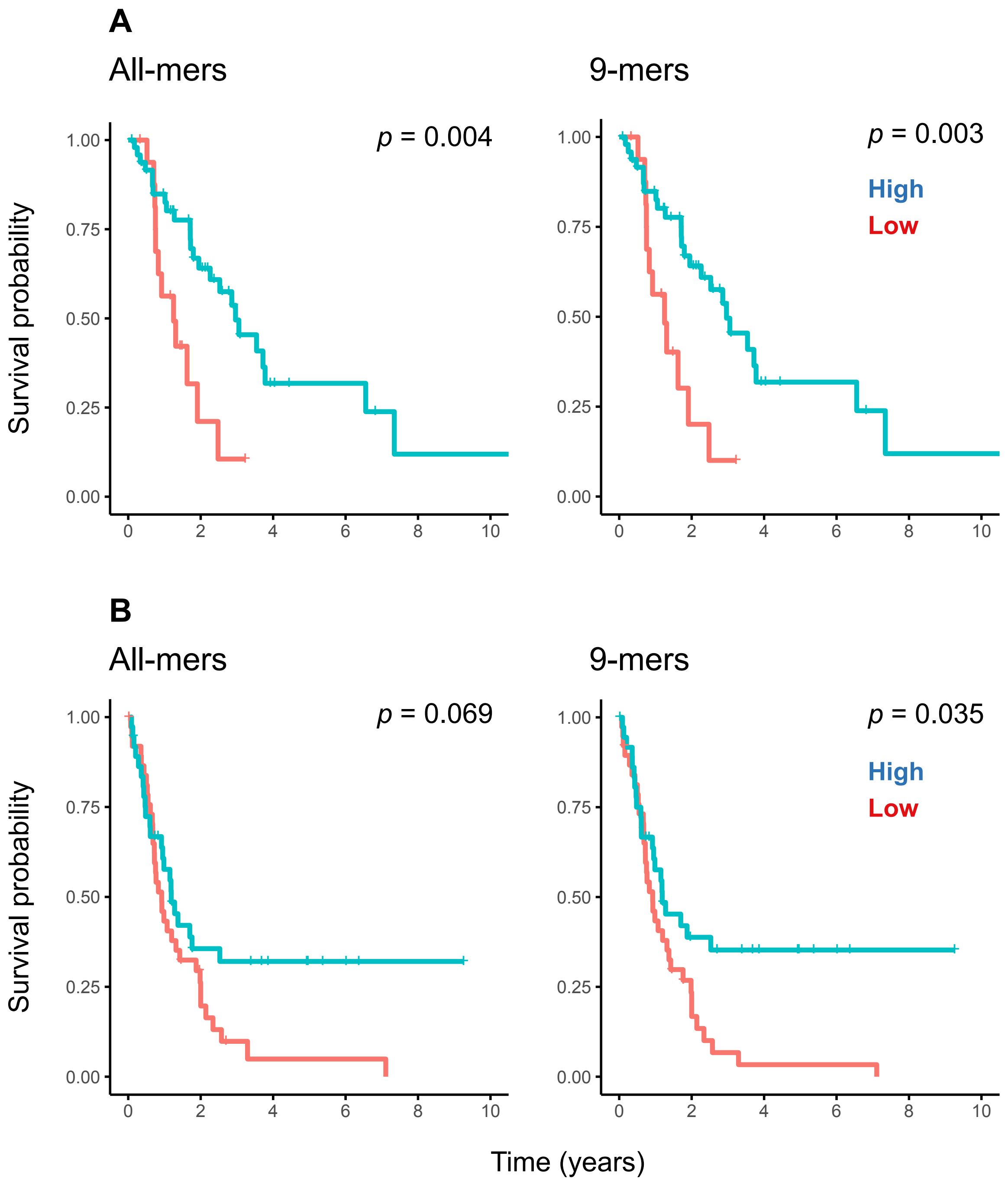

Kaplan-Meier survival curves for patients in the LUAD (A) and SKCM (B) cohorts comparing the association between survival and mean DAI (LUAD) and neoantigen mean DAI (SKCM) calculated using all-mers vs 9-mer predicted neopeptides. Stratification was done as per Figure 3 and log rank *p*-values are shown.
