# Supplementary material for: Differential binding affinity of mutated peptides for MHC class I is a predictor of survival in advanced lung cancer and melanoma
Source: Ann Oncol. 2017 Oct 23;29(1):271–9. doi: 10.1093/annonc/mdx687 (PMC5834109; doi:10.1093/annonc/mdx687)
Supplement: Supplementary Methods and Tables [file supplementary_methods_and_tables_mdx687.docx]

**Supplementary Data**

# Methods

## Neopeptide prediction and DAI analysis

Whole exome sequencing data for the clinical study cohorts was used in this analysis. For TCGA cohorts, clinical and RNA sequencing data were obtained through the Broad Institute FireBrowse portal ([www.firebrowse.org](http://www.firebrowse.org)) and single nucleotide variant data were obtained from TumorPortal [1].

Raw exome sequencing reads were aligned to the hg19 assembly and somatic variants identified. For each non-synonymous mutation, a sliding window approach was used to identify a list of all possible 9-11mer peptides and their binding affinities to the patient’s germline MHC-I alleles predicted using NetMHCpan-2.8. Samples with tumour and normal coverage of at least 50x and 30x respectively and with reliable copy number, mutation and purity estimation were included for analysis as previously published. Following exclusion of patients who did not meet these criteria, the final cohorts consisted of *n*=66 LUAD, *n*=75 SKCM, *n*=78 Van Allen [2], *n*=31 Rizvi [3] and *n*=51 Snyder patients [4].

In the original description of DAI, affinity prediction scores were generated using the NetMHC 3.0 Profile Weight Matrix algorithm [5]. In subsequent versions of NetMHC, artificial neural network based approaches have been adopted that generate predicted IC_50_ (nM) values [6] that were used here. To calculate DAI, MHC-I affinity was predicted for wildtype (WT_A_) and mutant (M_A_) peptide pairs arising from the same mutation and differing by a single amino acid. DAI was defined as WT_A_ - M_A_ for each peptide pair. In order to obtain a summary of the DAI for predicted peptides in each sample, the mean DAI was calculated. This was done based on the total number of mutated peptides predicted for each individual patient considering all peptides, those with binding affinity <500 nM and for those predicted to arise from clonal and subclonal mutations as previously defined [7].

To evaluate the relationship between peptide DAI and position of amino acid mutation, all 9mer peptides from the LUAD cohort were selected and DAI values predicted for HLA-A alleles. Peptides (n=166 746) were divided equally into DAI percentile groups, and within each group the proportion of peptides mutated at each position was calculated.

## Immune infiltration score analysis

In the SKCM cohort, pathological review of tissue specimens has previously defined a semi-quantitive score of lymphocyte infiltration (lymphocyte score; LS) based on cell density and distribution [8]. In the same report, unsupervised cluster analysis of the top 1500 expressed genes in 329 samples was used to define RNA expression subgroups corresponding to “immune-high”, “keratin-high” and “microphthalmia-associated transcription factor-low” categories. Patients were characterised into LS low (0-2) and high (3-6) subgroups as defined in the original report.

## MHC-II gene expression score and PDL1 expression status

A 13 gene MHC-II expression signature (comprising CD74, CIITA, CTSH, NCOA1 and the HLA genes HLA-DMA, -DMB, -DPA1, -DPB2, -DQA1, -DRB1, -DRB5, -DRB6) has previously been defined and found to correlate with immune infiltration in LUAD [9]. Upper-quantile scaled gene-level RNA-sequencing data was downloaded from the FireBrowse portal. For each patient, expression of the signature genes was log2 transformed to obtain normalised values and z-scores derived. The mean z-score was used as a measure of the MHC-II signature for each patient. Patients were grouped into MHC-II score low (<median expression) and high (>median expression) categories for analysis.

In the Rizvi [3] cohort, PDL1 expression was assessed by immunohistochemistry (IHC) as previously published. Patients were grouped by PDL1 expression into low (negative/weak) and high (strong) categories for analysis.

## Statistical analysis

Patients were initially categorised into mean DAI quartile groups for further analysis. Kaplan-Meier plots were used to visualise the survival distribution across quartiles and univariate Cox regression analysis was carried out using varying quartile thresholds to define low vs. high mean DAI patient subsets in the TCGA LUAD and SKCM cohorts. Thresholds defined at this stage (lower quartile cutpoint in LUAD and median cutpoint in SKCM) were subsequently used in analysis of independent clinical cohorts as a test of validity. Survival differences between groups were evaluated by log-rank test in univariate analysis and with multivariate Cox regression models.

Correlations between mean DAI and neoantigen burden, proportion of positive DAI peptides per patient (peptides with positive DAI/total predicted peptides) and maximum DAI were assessed by linear regression. Mean DAI was compared across cohorts by one way ANOVA and Tukey post-hoc analyses. The Wilcoxon rank sum test was used to evaluate the difference in mutational/NA burden and mean DAI by PDL1 expression and MHC-II score groups. All analyses were carried in the R environment with survival modelling carried out using the package survival. *P*-values of <0.05 was considered to indicate statistical significance and Benjamini-Hochberg correction was applied where indicated.

**References**

1. Lawrence MS, Stojanov P, Mermel CH et al. Discovery and saturation analysis of cancer genes across 21 tumour types. Nature 2014; 505(7484):495–501.

2. Van Allen EM, Miao D, Schilling B et al. Genomic correlates of response to CTLA-4 blockade in metastatic melanoma. Science (80-. ). 2015; 350(6257):207–211.

3. Rizvi NA, Hellmann MD, Snyder A et al. Mutational landscape determines sensitivity to PD-1 blockade in non-small cell lung cancer. Science (80-. ). 2015; 348(6230):124–8.

4. Snyder A, Makarov V, Merghoub T et al. Genetic Basis for Clinical Response to CTLA-4 Blockade in Melanoma. N. Engl. J. Med. 2014; 371(23):2189–2199.

5. Duan F, Duitama J, Al Seesi S et al. Genomic and bioinformatic profiling of mutational neoepitopes reveals new rules to predict anticancer immunogenicity. J. Exp. Med. 2014; 211(11):2231–48.

6. Andreatta M, Nielsen M. Gapped sequence alignment using artificial neural networks: application to the MHC class I system. Bioinformatics 2016; 32(4):511–517.

7. Mcgranahan N, Furness AJS, Rosenthal R et al. Clonal neoantigens elicit T cell immunoreactivity and sensitivity to immune checkpoint blockade. Science (80-. ). 2016; 351(6280):1463–1469.

8. The Cancer Genome Atlas Network. Genomic Classification of Cutaneous Melanoma. Cell 2015; 161(7):1681–1696.

9. Faruki H, Mayhew GM, Serody JS et al. Lung Adenocarcinoma and Squamous Cell Carcinoma Gene Expression Subtypes Demonstrate Significant Differences in Tumor Immune Landscape. J. Thorac. Oncol. 2017; 12(6):943–953.

# Tables

| **Table S1. Patient demographics** | | | | | |
| --- | --- | --- | --- | --- | --- |
|  |  |  |  |  |  |
| **Characteristic** | **LUAD (*n*=66)** | **Rizvi (*n*=31)** | **SKCM (*n*=75)** | **Van Allen (*n*=78)** | **Snyder (*n*=51)** |
| Age |  |  |  |  |  |
| Median | 67 | 63 | 57 | 63.5 | 63 |
| Range | 40–83 | 41–80 | 19–87 | 18–86 | 33–81 |
| Gender (*n*, %) |  |  |  |  |  |
| Male | 29 (44) | 16 (52) | 44 (59) | 57 (73) | 30 (59) |
| Female | 37 (56) | 15 (48) | 31 (41) | 21 (27) | 21 (41) |
| Stage IV (*n*, %) | 14 (21) | 31 (100) | 43 (57) | 69 (88) | 49 (96) |
| Mutations |  |  |  |  |  |
| Median | 122 | 65 | 164 | 165.5 | 287 |
| Range | 14–446 | 5–602 | 9–2200 | 8–1464 | 2–1557 |
| Neoantigens |  |  |  |  |  |
| Median | 231 | 90 | 388 | 398 | 630 |
| Range | 15–1006 | 3–1037 | 8–3409 | 12–5762 | 7–4110 |

| **Table S2. Mean DAI summary statistics** | | | | | | | |
| --- | --- | --- | --- | --- | --- | --- | --- |
|  |  |  |  |  |  |  |  |
| **Study** | **Min** | **Max** | **Median** | **Mean** | **SE** | **Range** | **IQR** |
| LUAD | 41.4 | 1115.8 | 595.2 | 606.7 | 27.1 | 1074.4 | 285.7 |
| Rizvi | 13.3 | 810.6 | 263.2 | 300.0 | 37.0 | 797.3 | 263.1 |
| SKCM | -208.5 | 1677.8 | 891.1 | 829.1 | 42.8 | 1886.3 | 353.1 |
| Snyder | -451.7 | 1637.7 | 893.4 | 844.7 | 51.1 | 2089.4 | 286.3 |
| Van Allen | -446.5 | 1528.7 | 874.5 | 823.6 | 39.8 | 1975.2 | 365.8 |
|  |  |  |  |  |  |  |  |
| Summary statistics for mean DAI of all peptides are represented. SE, standard error of the mean, IQR, interquartile range. | | | | | | | |

| **Table S3. Neoantigen mean DAI summary statistics** | | | | | | | |
| --- | --- | --- | --- | --- | --- | --- | --- |
|  | | | | | | | |
| **Study** | **Min** | **Max** | **Median** | **Mean** | **SE** | **Range** | **IQR** |
| SKCM | 276.6 | 4264.4 | 2225.9 | 2207.3 | 86.9 | 3987.8 | 921.5 |
| Snyder | 818.4 | 5657.0 | 2273.7 | 2389.3 | 114.4 | 4838.6 | 929.8 |
| Van Allen | 174.2 | 5497.1 | 2353.6 | 2352.7 | 94.1 | 5322.9 | 1140.1 |
|  | | | | | | | |
| Summary statistics for neoantigen mean DAI in melanoma cohorts are represented. SE, standard error of the mean, IQR, interquartile range. | | | | | | | |
